# CLIMP: Clustering Motifs via Maximal Cliques with Parallel Computing Design

**DOI:** 10.1371/journal.pone.0160435

**Published:** 2016-08-03

**Authors:** Shaoqiang Zhang, Yong Chen

**Affiliations:** 1 College of Computer and Information Engineering, Tianjin Normal University, Tianjin, China; 2 National Laboratory of Biomacromolecules, Institute of Biophysics, Chinese Academy of Sciences, Beijing, China; 3 Department of Biological Sciences, Center for Systems Biology, The University of Texas at Dallas, Richardson, Texas, United States of America; New York University, UNITED STATES

## Abstract

A set of conserved binding sites recognized by a transcription factor is called a motif, which can be found by many applications of comparative genomics for identifying over-represented segments. Moreover, when numerous putative motifs are predicted from a collection of genome-wide data, their similarity data can be represented as a large graph, where these motifs are connected to one another. However, an efficient clustering algorithm is desired for clustering the motifs that belong to the same groups and separating the motifs that belong to different groups, or even deleting an amount of spurious ones. In this work, a new motif clustering algorithm, CLIMP, is proposed by using maximal cliques and sped up by parallelizing its program. When a synthetic motif dataset from the database JASPAR, a set of putative motifs from a phylogenetic foot-printing dataset, and a set of putative motifs from a ChIP dataset are used to compare the performances of CLIMP and two other high-performance algorithms, the results demonstrate that CLIMP mostly outperforms the two algorithms on the three datasets for motif clustering, so that it can be a useful complement of the clustering procedures in some genome-wide motif prediction pipelines. CLIMP is available at http://sqzhang.cn/climp.html.

## Introduction

The rapid development of new technologies has led to the declining cost of genome sequencing, and as a result, thousands of genomes are being sequenced [1, 2]. Furthermore, numerous comparative genomics-based algorithms have been developed in order to decipher the biological functions of various sequenced genomes; this can be computed because these biological functions are encoded and relatively conserved in a group of closely related genomes. Moreover, transcription regulation is usually triggered by the binding of proteins called transcription factors (TFs) to specific DNA segments known as TF binding sites (TFBSs). Furthermore, these TFBSs are for the most part predicted by comparing multiple non-coding sequences that potentially contain the TFBSs. A set of TFBSs recognized by the same TF is called a motif, which summarizes the commonalities among the binding sites of a TF [3]. Additionally, numerous motif-finding algorithms have been designed to identify overrepresented segments of sequences as potential TFBSs from a set of regulatory regions of some co-regulated genes with the advent of gene expression profiling technologies (e.g. DNA microarray, SAGE, Tiling array, and the latest popular RNA-Seq technology [4]) [5–7]. Based on the observation that a particular TF’s binding sites are relatively conserved in a set of closely related genomes, various algorithms using the phylogenetic foot-printing technique have been proposed so as to identify conserved DNA segments as potential TFBSs from the promoters of orthologous genes in a group of related genomes.

In the last few years, with the development of the next-generation sequencing (NGS) technologies, more and more genome-wide profiling data of DNA binding proteins are provided by the ChIP-chip and ChIP-Seq techniques [8–10]. In a TF ChIP dataset, its binding sites are highly enriched. However, the sequenced segments in a ChIP dataset are much longer than the ChIP-ed TF binding sites, so peak-calling tools can be employed to identify the binding peaks in the potential binding regions to cut down each segment to hundreds or thousands of base pairs (bp) [11]. Then for the constricted regions of the ChIP-ed TF, motif-finding tools are used to identify its corresponding motifs [12]. Therefore, if all known TFs in a genome have been ChIP-ed, a lot of motifs of these TFs and their co-factors can be predicted [13].

Frequently, after a certain amount of new putative motifs are obtained, the next step becomes separating real motifs from spurious ones and clustering real ones into groups so that grouped motifs belong to the same TF, and accordingly, different groups correspond to different TFs. Therefore, two developments are desired: first, a novel metric for measuring the similarity between two motifs and, second, an efficient clustering algorithm for merging motifs of the same TF family. Many metrics have been proposed for motif comparison. For example, the sum of squared distances [14, 15], the *p*-value of Chi-square [16], the average log-likelihood ratio [17], the average Kullback-Leibler [18], Pearson’s correlation coefficient (PCC) [19], Asymptotic Covariance [20], the *k*-mer frequency vector [21], and SPIC [22] have been used for computing similarities between motifs. A web server STAMP has been built via integrating the first five metrics and alignment algorithms after assessing them [23, 24]. Recently, all of the eight metrics were assessed, and the metric SPIC was shown to outperform the others in separating relevant motifs from spurious ones [22]. Now, an efficient clustering algorithm is required for grouping motifs so as to separate real motifs from spurious ones.

Genome-wide motif similarity data are generally represented as a large network or graph, where great quantities of motifs are joined to one another. In the graph, each node represents a motif, and the weight of each edge that joins two nodes represents the similarity between the corresponding two motifs. A clustering procedure, which aims to identify densely connected sub-graphs in the similarity graph, is commonly used to group such motifs. Many graph clustering algorithms have been developed, and a select few have been applied to the motif clustering problem. Among these algorithms for motif clustering, the most successful one is the MCL (Markov Clustering) algorithm [25], which was shown to outperform some other clustering algorithms (i.e., Bayesian clustering [26], Monte Carlo sampling [27], and PhyloNet [15]) in the references of GLECLUBS [28] and eGLECLUBS [29] as well as in some applications for partitioning protein interaction graphs [30, 31]. About a year ago, Niu *et al*. [13] designed a tool called DePCRM to predict binding sites and *cis*-regulatory modules of *D*. *melanogaster* through integrating a large number of ChIP datasets; and MCL was expropriated to cluster motifs predicted by a motif finding tool. The MCL algorithm simulates random walks and alternately runs expansion and inflation operations on a node-similarity graph represented by a “Markov” matrix, each of whose entries represents the transition probability between a pair of nodes. Moreover, AP (Affinity Propagation) [32] is another famous clustering algorithm that has been used to cluster protein interaction networks [31] and detect genes in microarray data [32].

A survey on graph clustering summarized by Schaeffer [33] classified graph clustering methods into three categories: density-based methods (e.g. *k*-means and *k*-center [34]), cut-based methods (e.g. hierarchical clustering [35] and spectral clustering [36]), and random walks methods (e.g. MCL). In fact, the main aims of density-based and cut-based methods are to find maximum cliques and sparsest cuts, respectively, both of which are known to be NP-hard [37, 38]. Methods, such as *k*-means, k-center, and expectation maximization (EM) clustering [39] algorithms, keep a relatively small set of estimated cluster centers at each step. Afterwards, AP improves these algorithms by simultaneously considering all data points as candidate centers and then gradually merging them to identify clusters. It should also be noted that both hierarchical clustering and spectral clustering are only capable of dealing with another type of clustering problem of recursively comparing pairs of data points to partition the data. However, hierarchical clustering and spectral clustering methods, are not well-suited to group motifs because two motifs that should not be clustered together may, in fact, be clustered together by a series of pairwise groupings [32]. These applications for both motif similarity graphs and protein interaction graphs encounter the additional problem of not properly managing a significant level of background noise (e.g. the high similarity score among spurious motifs). Therefore, in this paper, AP and MCL, which are the best as we know among these density-based and random walks methods respectively, are selected to make comparisons.

Due to the large-scale collection of similarity data among a large number of motifs produced from genome-wide prediction as well as the additional problem of handling background noise, a clustering algorithm that can tolerate mass data is required in order to produce more accurate results in a short timespan than what is produced by other existing methods. Therefore, in this paper, we propose a new clustering algorithm called CLIMP (Cluster Cliques of Motifs in Parallels with openMP) and demonstrate that it mostly outperforms two outstanding clustering algorithms, MCL and AP, for large-scale motif similarity graph clustering.

## Methods

### Motivation and basic idea

The binding sites belonging to a TF may be identified by one or more motif finding tools in one or more datasets. Motif finding tools are usually designed for finding well-conserved sites in the upstream sequence set of either a group of co-regulated genes in a genome or a group of orthologous genes in a set of closely related genomes (i.e., the phylogenetic foot-printing technique), or in a ChIP dataset of a TF. The binding sites of a TF are often degenerate in a genome and divergent across related genomes [26, 40, 41]. Due to the degeneration and diversity of the binding sites of a TF, multiple distinct sub-motifs of the TF may be found by one motif finding tool through outputting multiple top results or by multiple motif finding tools. For example, the experimentally verified motif of the TF CRP in *E*. *coli* K12 can be classified into at least three well-conserved sub-motifs; i.e., a canonical palindromic sub-motif, an A-rich sub-motif, and a T-rich sub-motif although both the latter ones share a certain number of elements with the canonical one [28]. A report showed that roughly half of 104 distinct mouse DNA binding proteins each recognized multiple distinctly different sequence motifs when the binding sites were examined in the mouse ChIP-chip datasets [41]. Furthermore, the binding sites of some TFs were reported to be always divergent in three different yeast species (*S*. *cerevisae*, *S*. *mikatae*, and *S*. *bayanus*) [40]. For example, of the 221 and 255 recognized sites bound in total by two TFs Ste12 and Tec1, respectively, only 47 (Ste12, 21%) and 50 (Tec1, 20%) sites were conserved across all three yeast species [40]. So a certain percentage of Ste12 and Tec1’s sites were conserved across at most two yeast species. Suppose that the entire motif *M* of a TF can be divided into *k* well-conserved sub-motifs {*M*_1_, *M*_2_, …, *M*_*k*_}. If each well-conserved sub-motif *M*_*i*_ is partially or fully predicted many times by one or more motif-finding tools from multiple sequence sets, a set *P*(*M*_*i*_) of predicted motifs corresponding to each sub-motif *M*_*i*_ will be found. If each predicted motif of a sub-motif is treated as a node of a graph, and two predicted motifs are connected by an edge if their similarity is above a cutoff value, the predicted motif set *P*(*M*_*i*_) of a sub-motif *M*_*i*_ are likely to form a clique (i.e., a complete subgraph). Therefore, a sub-motif can be modelled as a clique of its predicted motifs and a motif composed of *k* sub-motifs can be modelled as the mergence of *k* cliques.

### The CLIMP algorithm with parallel computing design

For a set of binding site motifs with their corresponding position frequency matrices and position weight matrices, a motif similarity graph is constructed by using the SPIC metric to compute the similarity score between each pair of motifs. In the graph, each node represents a motif, and two nodes are connected by an edge, whose weight is the similarity score between the corresponding two motifs, if and only if the similarity score is greater than a preset threshold. More specifically, binding site motifs that belong to the same TF are more likely to form highly connected sub-graphs with high edge weights in the motif similarity graph than are those from different TFs or spurious motifs. However, due to the degenerate nature of the binding sites from the same motif, the similarity between two subsets (called sub-motifs, here) of a motif may not be significantly high. For this reason, motifs that are very similar to each other are initially grouped together in order to generate a set of clusters, and then, each of the remaining motifs is assigned into a cluster if the motif is similar to a large proportion of the motifs present in a given cluster. Given a motif similarity graph *G* = (*V*, *E*) where *V* is the set of nodes and *E* is the set of edges, the algorithm is separated into four steps as follows.

**Step 1:** *For each node*, *find a maximal clique associated with it*. For a node, a “greedy” strategy is used to find a maximal clique associated with the node. The clique can be regarded as the cohesion of the node.

The problems of enumerating all the maximal cliques and finding the maximum clique in a graph are NP-hard [38]. Here, only |*V*| maximal cliques rather than all the maximal cliques and the maximum clique in the graph *G* = (*V*, *E*) would be intended to be found, where |*V*| is the number of nodes in *G*. That is, for each given node *v*, it is intended that a maximal clique would be found, whose nodes have the closest relationship to *v*. For this purpose, the neighborhood sub-graph *N*(*v*) of a node *v* is defined as the sub-graph induced by *v* and its neighbor nodes. Definitely, all the maximal and maximum cliques containing the node *v* are in *N*(*v*). Here, for each node *v*, the neighborhood sub-graph *N*(*v*) is extracted from the graph *G* and a greedy strategy to find a maximal clique *C*_*v*_ in *N*(*v*) is designed as follows:

1. Set *C*_*v*_ = *N*(*v*). If *C*_*v*_ is a clique, stop; else the neighbor nodes of *v* are sorted in ascending order by the weights of their edges incident to *v* to get an array *U* = {*u*_1_,*u*_2_,…,*u*_*t*_}. Go to (b).
2. For each node *u*_*i*_ in the array *U*, *u*_*i*_ and its incident edges are sequentially deleted until the degrees of *v* and the remaining nodes are identical in *C*_*v*_. The deleted nodes are labelled as an array {*u*_1_,…,*u*_*k*−1_,*u*_*k*_}. Go to (c).
3. For each node *u*_*j*_ from *u*_*k*−1_ to *u*_1_ in reverse order, if each of the nodes in *C*_*v*_ is joined with *u*_*j*_ by an edge in *N*(*v*), update *C*_*v*_ by adding *u*_*j*_ and its incident edges {(*u*_*j*_,*u*):*u* ∈ *C*_*v*_} into *C*_*v*_. Stop.

**Fig 1:**
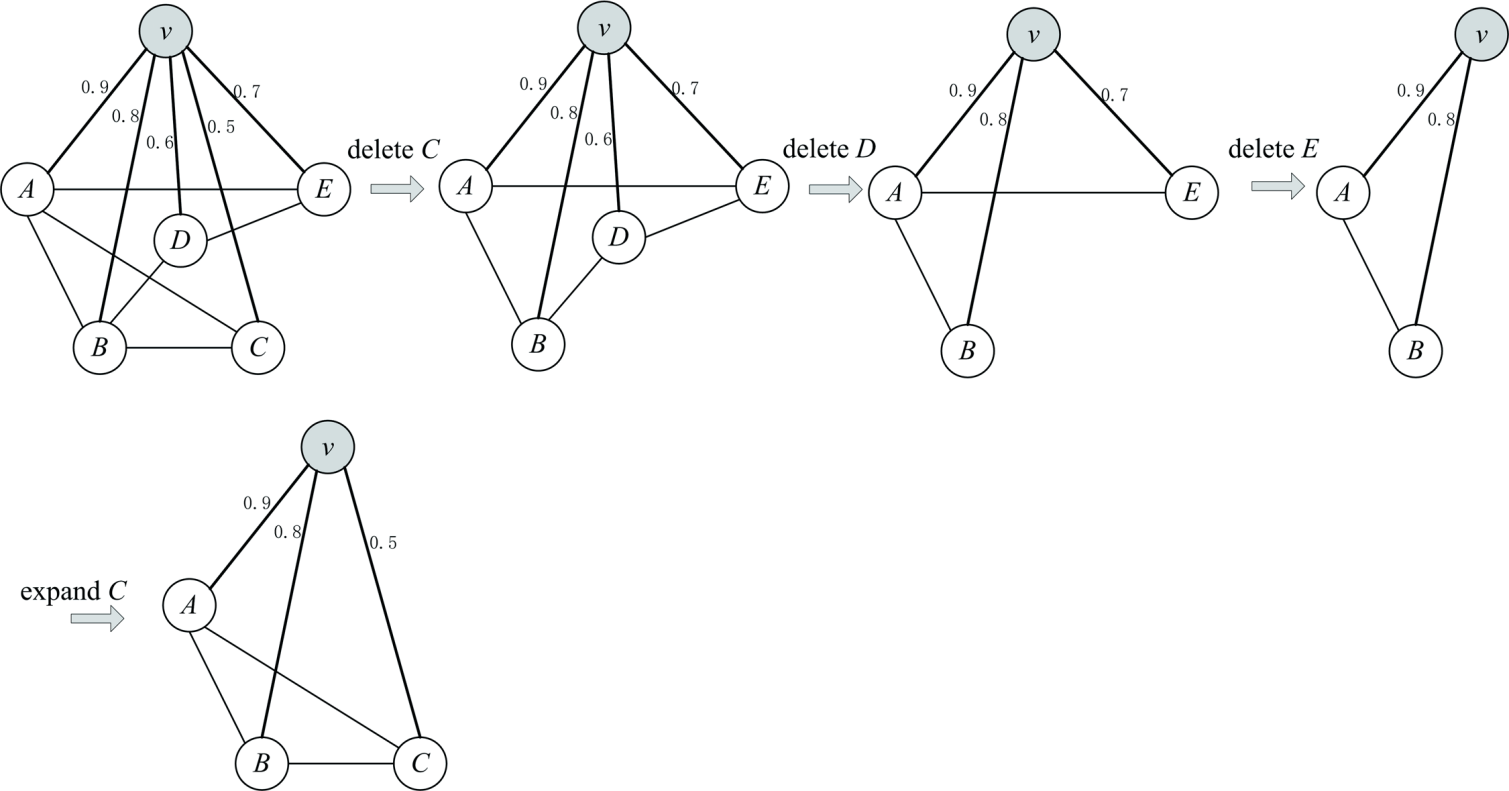
An example of finding a maximal clique associated with node *v* in *N*(*v*). (a) Sort the neighbor nodes to get an array {*C*, *D*, *E*, *B*, *A*}. (b) Successively delete the nodes *C*, *D*, and *E* as well as their incident edges from *N*(*v*) to get *C*_*v*_ until *v* and the remaining nodes have the same degree. (c) For *D* and *C*, determine if they can be expanded to *C*_*v*_.

The finally obtained *C*_*v*_ is called the clique associated with node *v*. An example of finding a maximal clique associated with node *v* in sub-graph *N*(*v*) is illustrated in Fig 1. It should be noted that the clique that is obtained in this step may not be the maximum clique associated with *v* in *N*(*v*). Even though the obtained maximal clique may not be a maximum clique, it remains superior to the maximum clique because its nodes have the closer relationship (higher similarity) to *v* than do the nodes in the maximum one.

For each node *v*, the procedure for finding its associated clique is identical, and the time complexity is $O(d_{v}^{2})$ where *d*_*v*_ is its degree. Fortunately, the motif similarity graph is generally sparse, and the degree *d*_*v*_ is usually small. In this step, the “for” loop for clique finding can be easily parallelized. For example, if the openMP libraries (http://openmp.org) are included in the program, the routine, “*#pragma omp parallel for*” is just called before the “*for*” loop. If *k* processes are invoked simultaneously, the time complexity will be reduced to $O(|V|\cdot \max_{v\in V}\{d_{v}^{2}\}/k)$, where |*V*| is the number of nodes in graph *G* = (*V*, *E*).

**Fig 2:**
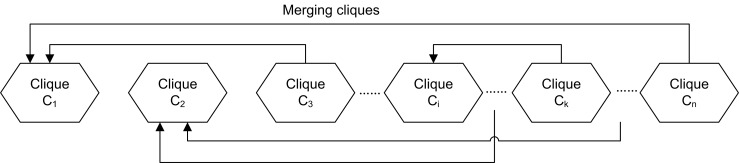
An illustration of merging cliques.

**Step 2:** *Merge cliques into clusters*. Based on the law of gravity, for two substances, a third has greater attraction with the heavier one of them if the third has the same distance from the two substances. Similarly, for two cliques, a third clique has a greater affinity with the bigger one of them if the third has the same extent of overlap with the two cliques. So a large clique is more likely to be the core of a cluster than a small one is. In other words, the smaller a clique is, the more likely it is that its nodes do not belong to the same cluster. Therefore, in this clique merging step, both the sizes of cliques and the extent of overlapping among cliques are considered. Initially, all redundant cliques are merged to form unique ones, and then, all unique cliques are sorted in descending order by the sum of edge weights in order to generate a ranked queue {*C*_1_,*C*_2_,…,*C*_*n*_}. Subsequently, as shown in Fig 2, the procedure begins from the first largest clique, and for each current unassigned clique, it is set as an initial cluster. Then, any following unassigned cliques are successively integrated into the cluster if an unassigned clique has a significant number of duplicated nodes with the current clique. The procedure is finished after all of the cliques in the queue are assigned.

For two cliques *C*_*i*_ and *C*_*j*_ (*i* < *j*), what is the specific rule of merging *C*_*j*_ into *C*_*i*_’s cluster? If the (overlap) ratio of the nodes in *C*_*j*_ appearing in *C*_*i*_ is no less than *α* (i.e., |*C*_*i*_∩*C*_*j*_|/|*C*_*j*_|≥*α*) and in the graph *G* the ratio of nodes of *C*_*j*_ having adjacent nodes in *C*_*i*_ is no less than *β*, *C*_*j*_ is merged into *C*_*i*_’s cluster; otherwise, nothing is done. Such a process is labeled as $\left(C_{i}\overset{?}{\leftarrow }C_{j}\right)$ for a pair of cliques *C*_*i*_ and *C*_*j*_ (*i* < *j*). The parameters *α* and *β* (*α* ≤ *β*) can be set by users.

**Fig 3:**
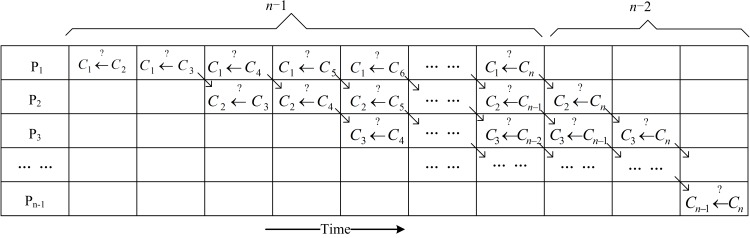
Pipeline of space-time diagram.

This step can also be parallelized by using a pipeline design. For each clique *C*_*i*_ from *i* = 1 to *n*−1, a different processor can be called to run the merging processes $\left(C_{i}\overset{?}{\leftarrow }C_{j}\right)$ from *j* = *i*+1 to *n*. As shown in Fig 3, after running a process $\left(C_{i}\overset{?}{\leftarrow }C_{j}\right)$ in processor *P*_*i*_, if *C*_*j*_ cannot be merged into *C*_*i*_, the process $\left(C_{i+1}\overset{?}{\leftarrow }C_{j}\right)$ in processor *P*_*i*+1_ and the process $\left(C_{i}\overset{?}{\leftarrow }C_{j+1}\right)$ in processor *P*_*i*_ are run simultaneously. Clearly, if only one processor is called, the total number of processes is no more than 1 + 2 + ⋯ + (*n*−1) = (*n*−1)*n*/2, but if *n* processors are simultaneously called, the asynchronous processes are at most (*n*−1) + (*n*−2) = 2*n*−3, as illustrated in Fig 3.

**Step 3:** *Delete redundant nodes*. In the reduced sub-graph of a cluster, for each node the corresponding weight sum of edges incident to it in the sub-graph is first calculated. For all clusters, because there is no interaction between each pair when the edge-weight sums of each node is calculated, this step can be parallelized by separately dealing with the clusters in different processors. After that, for each node that appears redundantly in more than one cluster, only that which has the maximum edge-weight sum is kept, and the redundancies are deleted from these clusters.

**Step 4:** *Sort clusters*. All clusters are sorted in a descending order of edge-weight sums in order to obtain the final set of ranked clusters. Note that the calculation of each cluster’s edge-weight sum can also be parallelized.

Among the four steps described in the CLIMP algorithm, the largest computation involves finding all maximal cliques in Step 1. Since motif similarity graphs are generally sparse and there is no vector-vector or matrix-matrix multiplication in clique finding, an adjacency list is used to store such a sparse graph instead of an adjacency matrix in order to reduce graph *G’*s storage. In Step 1, for each node *v*, only a list of its neighbors is required to be reported in *O*(|*d*_*v*_|) time, and the neighbors are represented as a sorted array according to edge weight. Finally, the pseudo-code of the parallel clustering algorithm is shown in Table 1.

**Table 1:** The pseudo-code of CLIMP.

1. Input: Similarity graph *G* = (*V*,*E*);
2. Output: A set of clusters;
3. Parameters: α, β, γ (γ is the cutoff of motif similarity), and number of threads.
4. For (node i = 1. n with step i: = i+1)
5. Open a thread P_i_ to find a maximal clique C_i_ associated with i;
6. End for
7. Sort Cliques(C_1_, C_2_, …, C_n_);
8. For each C_i_ (i = 1. n-1 with step i: = i+1) in a parallel pipeline
9. Open a thread P_i_;
10. If C_i_ is not labeled “merged”, in the thread P_i_
11. For each C_j_ (j = i+1. n with step j: = j+1)
12. If C_j_ is not labeled “merged”
13. If |*C*_*i*_∩*C*_*j*_|/|*C*_*j*_|≥*α* and the ratio of nodes of C_j_ having adjacent nodes in C_i_ is no less than β
14. Merge C_j_ into C_i_ and label C_j_ “merged”; (i.e. the process C_i_←C_j_)
15. Else if C_i+1_ is not labeled “merged”
16. In the thread P_i+1_ to do the process C_i+1_←C_j_;
17. End if
18. End if
19. End for
20. End if
21. End for
22. Delete redundant nodes;
23. Sort clusters.

### Performance assessment

Clearly, an ideal motif clustering algorithm can group two relevant motifs in a cluster in addition to separating two irrelevant motifs in different clusters. In a perfect motif clustering result, each cluster should contain exactly one motif, and each motif should also only be located in exactly one cluster. For *m* obtained clusters and *n* given motifs, the ability of a clustering algorithm to recover motifs from a motif similarity graph is evaluated using the Adjusted Rand Index (ARI) [42] derived from a contingency table (*n*_*ij*_)_*n*×*m*_, where each *n*_*ij*_ represents the number of objects that are in both motif *i* and cluster *j*. Let *N* be the number of all objects. Let *n*_*i*•_ and *n*_•*j*_ be the number of objects in motif *i* and cluster *j*, respectively. The formula of the Adjusted Rand Index is:

$$ARI=\frac{\sum_{i,j}\left(\begin{array}{c} n_{ij}\\ 2 \end{array}\right)-\left[\sum_{i}\left(\begin{array}{c} n_{i•}\\ 2 \end{array}\right)\sum_{j}\left(\begin{array}{c} n_{•j}\\ 2 \end{array}\right)\right]/\left(\begin{array}{c} N\\ 2 \end{array}\right)}{\frac{1}{2}\left[\sum_{i}\left(\begin{array}{c} n_{i•}\\ 2 \end{array}\right)+\sum_{j}\left(\begin{array}{c} n_{•j}\\ 2 \end{array}\right)\right]-\left[\sum_{i}\left(\begin{array}{c} n_{i•}\\ 2 \end{array}\right)\sum_{j}\left(\begin{array}{c} n_{•j}\\ 2 \end{array}\right)\right]/\left(\begin{array}{c} N\\ 2 \end{array}\right)}.$$

### Programs and parameter optimization

The MCL program used in the paper was released on May 17, 2014 (http://micans.org/mcl/src/mcl-14-137.tar.gz). The AP program was downloaded from Frey Lab’s homepage (http://www.psi.toronto.edu/affinitypropagation/software/apcluster_linux64.zip). All three clustering programs (MCL, AP, and CLIMP) were compiled and installed on 64-bit Linux (x86_64). To ensure a comparison that is as fair as possible among the three clustering algorithms, the values of the adjustable parameters in CLIMP, MCL, or AP were selected so as to maximize the Adjusted Rand Index. For the MCL algorithm, we sampled the values of the Inflation parameter from 1.5 to 4.0 in steps of 0.1. For AP, the values of the Reference parameter were sampled from 0.1 to 1.0 in steps of 0.05. The number of iterations with no change in the clusters that stop the convergence was set to 1500, the number of maximum iterations to 200,000, and the damping factor to 0.99. For the CLIMP algorithm, the values of the parameters *α* and *β* were sampled from 0.1 to 0.9 in steps of 0.1, respectively, to form all possible combinations that satisfy *α* ≤ *β*. Given the Position Weight Matrices (PWMs) and Position Frequency Matrices (PFMs) of two motifs *M*_1_ and *M*_2_, the SPIC metric first uses *M*_1_’s column information contents as a factor to compute the likelihood of *M*_1_’s PWM generating *M*_2_’s PFM and vice versa, then averages the two likelihood values. The SPIC program including an example was downloaded from Zhang’s homepage (http://bioinfo.uncc.edu/szhang/app/spic.zip) and the similarity cutoff values were sampled from 0.4 to 0.7 in steps of 0.05.

## Results and Discussion

### Parameter selection and performance on motif retrievals

Currently, all motif-finding tools are limited as they can only find the partial binding sites of a TF; consequently, all TF binding sites always appear as subsets of them. Furthermore, any two subsets (sub-motifs) of binding sites recognized by the same TF are always highly conserved. Therefore, a perfect motif clustering algorithm should be able to ensure that each cluster only contains the binding site motifs of exactly one TF as well as locate each TF’s motifs in exactly one cluster. Therefore, if the binding sites of a TF are shuffled to generate a series of sub-sets (sub-motifs), a clustering algorithm is necessarily proposed to test whether these sub-motifs can then be clustered together again. In order to estimate the parameters of CLIMP and evaluate CLIMP’s performance on grouping sub-motifs from the same motifs together and separating sub-motifs from different motifs, all non-redundant transcription factor binding sites (TFBSs), which belong to 593 motif profiles, were first downloaded from the JASPAR core database Version 5.0 (http://jaspar.genereg.net/html/DOWNLOAD/sites.tar.gz), which is a collection of experimentally defined TFBSs for eukaryotes [43]; and these motifs are then used to generate numerous sub-motifs. We used the method described in the paper for evaluating the SPIC metric [22] to produce artificial sub-motifs. For each motif consisting of *n* TFBSs, the motif is randomly divided into two sub-sets (sub-motifs) of sizes *k* and *n*−*k*, respectively, for each *k* = 1,2,…,[*n*/2]. Therefore, 2×[*n*/2] sub-sets (sub-motifs) can be generated for a motif of *n* TFBSs. Moreover, it is obvious that a motif with a greater number of binding sites would necessarily result in a greater number of sub-motifs. In addition, since all motif-finding tools were designed to find overrepresented segments as predicted binding sites in a set of DNA sequences, an “overrepresented” motif (i.e., a motif that has more binding sites) is more easily distinguished by motif-finding tools than a “non-overrepresented” motif (i.e., a motif that has fewer binding sites). Therefore, a motif of *n* binding sites is divided into about *n* sub-motifs. For the 593 motif profiles, 30,000 sub-motifs are finally obtained. For these sub-motifs, the SPIC metric is employed to calculate the similarity between each pair.

**Fig 4:**
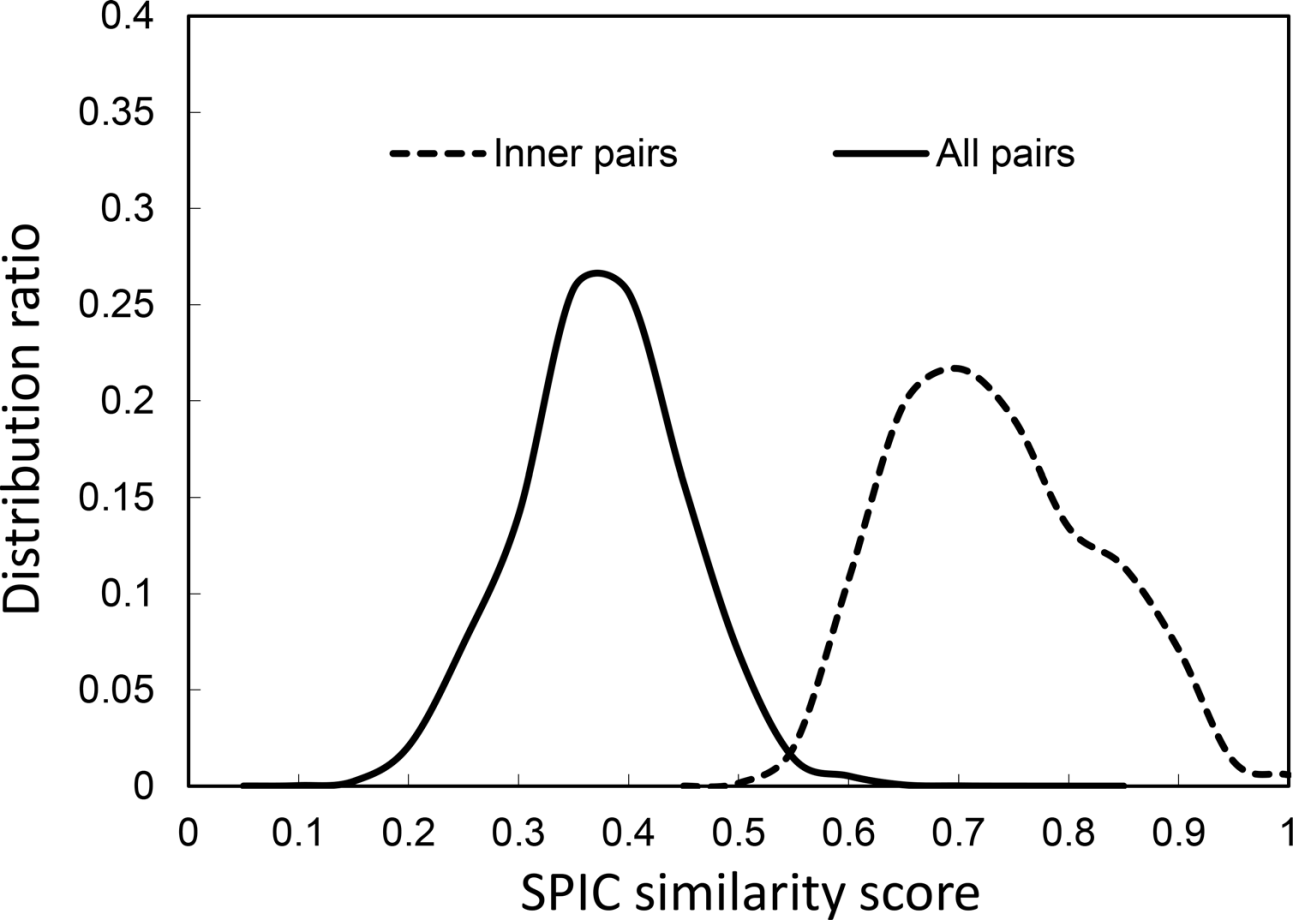
The distributions of motif similarity scores as computed by SPIC metric.

In addition, two distributions (Fig 4) are plotted in order to determine whether the similarity graph contains clustering properties. In Fig 4, the curve labeled as “all pairs” is the distribution of the similarity scores between each pair of the 30,000 sub-motifs, and the curve labeled as “inner pairs” is the distribution of the similarity scores between each pair of sub-motifs within the same profile. Clearly, the two curves have a small overlapping area. Based on Fig 4, a similarity score cutoff can be chosen such that as many as possible nodes that represent the sub-motifs of a particular motif profile are connected, while as many as possible nodes that represent sub-motifs of different motif profiles are disconnected. Therefore, the SPIC-constructed similarity graph will have the sparsest edges, whereas the relevant sub-motifs are still likely to be connected if the similarity score cutoff *γ*>0.4 as shown in Fig 4. For example, even if *γ* = 0.6, 83% of the sampled sub-motifs of a motif profile had an “inner pair” similarity score greater than 0.6, and the graph constructed with 0.6 as the cutoff contained only 1.3% of “all pairs” possible edges of the motif similarity graph.

**Fig 5:**
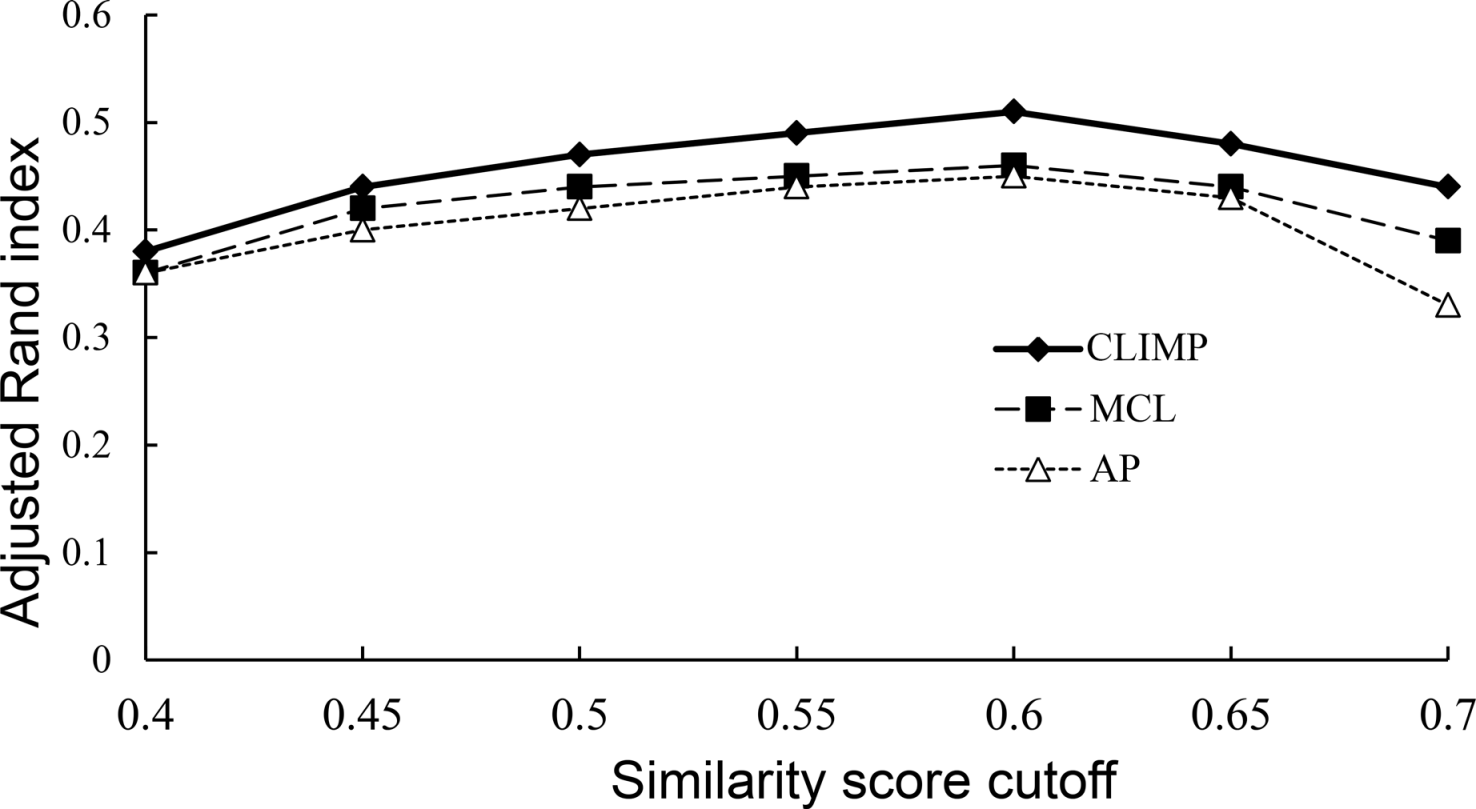
ARI values. The adjusted Rand index values at different motif similarity cutoffs for the three clustering algorithms.

After the construction of a motif similarity graph, the clusters produced by each of the three clustering tools in sub-motif similarity graphs with different cutoff settings are evaluated. Based on the observation of Fig 5, the optimal similarity score cutoff falls within the range [0.4, 0.7]. From *γ* = 0.4 to 0.7 with an interval of 0.05, sub-motif similarity graphs are successively constructed by keeping all edges with weights of no less than *γ*. Each of the three tools are used on each graph with their optimal parameters in order to acquire a set of clusters, and the corresponding adjusted Rand index values are calculated. As shown in Fig 5, each of the three clustering algorithms achieves the highest ARI values at the 0.6 cutoff, and of important notice, CLIMP outperforms both MCL and AP in these graphs with different cutoffs for clustering sub-motifs of the same motif and separating sub-motifs belonging to different motifs.

When the similarity score cutoff is 0.6, MCL, AP, and CLIMP are separately used to cluster the similarity graph with their optimal parameters (i.e., the Inflation parameter value of MCL is 2.6, the Reference parameter value of AP is 0.55, and (*α*, *β*) = (0.5, 0.5) for CLIMP), which can maximize their adjusted Rand indices. Finally, 1647, 1423, and 1569 clusters are respectively output by CLIMP, MCL, and AP. Clearly, a perfect clustering solution should result in one cluster corresponding to one motif. To evaluate the correspondence of the motif profiles and the clusters obtained by each tool, the number of motif profiles recovered by a cluster was first counted. From which, the majority of them corresponded to exactly one motif profile. For CLIMP, 62% of the clusters each contain only one motif profile, while the percentage is 56% in the MCL’s clusters and 51% in the AP’s clusters. Conversely, the number of obtained clusters that each motif profile’s sub-motifs are located in was also counted. The majority of the 593 known motif profiles were clustered into one cluster. For CLIMP, 45% of the motifs were located in exactly one cluster, while the corresponding percentages are 47% and 48% for MCL and AP, respectively.

### Performance on identifying true motifs from putative motifs

A genome-wide phylogenetic foot-printing dataset of yeast was downloaded from MotifClick’s website (http://motifclick.uncc.edu/yeast_intergenic_seq_sets.tar.gz) [44]. The dataset is composed of 5,137 intergenic sequence sets of orthologous genes from the target genome *Saccharomyces* (*S*.) *cerevisiae* and 6 reference genomes (*S*. *castellii*, *S*. *bayanus*, *S*. *kluyveri*, *S*. *mikatae*, *S*. *kudriavzevii*, and *S*. *paradoxus*). More specifically, orthologous genes between two genomes were predicted by the bidirectional best hits (BDBH) method using BLASTP with an E-value cutoff of 10^−20^ for both searches. Then, for each group of orthologous genes in the seven genomes, up to 1,000 bases upstream inter-genic region of each gene were extracted to form an orthologous sequence set. Finally 5,137 orthologous sequence sets each containing at least three sequences were obtained. As illustrated in the MotifClick paper [44], the motif length is set as eight bases, and three performance-outstanding motif-finding tools, MotifClick [44], MEME [45], and BioProspector [46], are separately run in the ‘anr’ mode if available to output the top 10 motifs on each of the 5,137 sequence sets. As a result, approximately 150,000 putative motifs, which contain 122 known TF motifs of *S*. *cerevisiae* in both the YEASTRACT database (http://www.yeastract.com/download/TFConsensusList_20130918.Transfac.gz) [47] and the *Saccharomyces* Genome Database (SGD) (http://downloads.yeastgenome.org/published_datasets/MacIsaac_2006_PMID_16522208/) [48], are obtained. Based on the fact that a TF can regulate multiple genes and a real motif is more likely to be predicted by multiple motif-finding tools than any spurious one, a real motif belonging to the same TF could be gathered in a set of similar putative predicted motifs. MCL, AP, and CLIMP are then tested to cluster these putative motifs to see whether or not the clusters that contain a majority of the 122 known motifs rank high on the cluster list.

**Fig 6:**
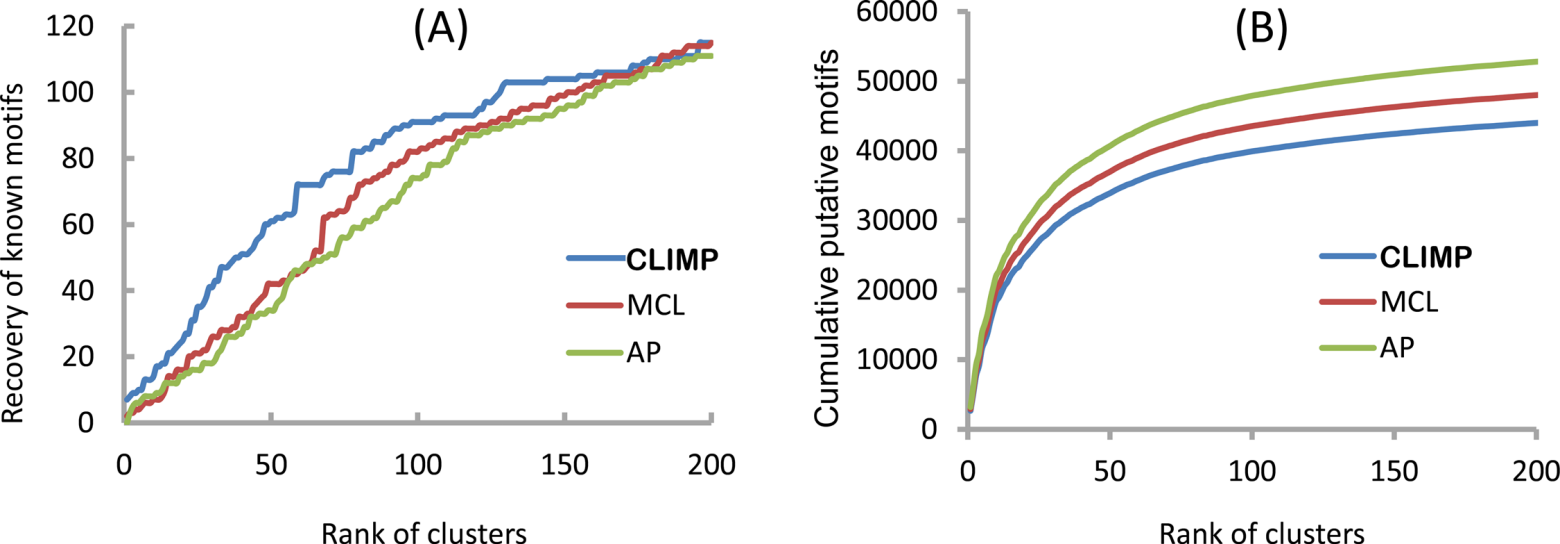
Evaluation of the three clustering algorithms in a phylogenetic foot-printing dataset. Cumulative numbers of recovered known motifs (A) and putative motifs (B) of the yeast phylogenetic foot-printing dataset in the top-ranked clusters produced by MCL, AP, and CLIMP, respectively.

At first, the SPIC metric is utilized to compute the similarity between each pair of these putative motifs, and the cutoff is chosen as 0.6 based on the analysis in the first experiment in order to generate a motif similarity graph with 145,581 nodes and 34,413,340 edges. The three clustering algorithms with their optimal parameters in the first experiment (i.e., the Inflation parameter value of MCL is 2.6, the Reference parameter value of AP is 0.55, and (*α*, *β*) = (0.5, 0.5) for CLIMP) are successively run on the resulting motif similarity graph. We say a putative motif recovering a true motif if the sites of the target genome in the putative motif are binding sites of the true motif. As shown in Fig 6(A), the top 130 clusters of CLIMP recover 104 (85.2%) of the total 122 motifs, whereas the top 130 clusters of MCL and AP only recover 92 (75.4%) and 90 (73.8%) of the 122 motifs, respectively. After the 130th cluster, the motif recovery rate of CLIMP’s clusters increases at a more gradual rate than do MCL’s and AP’s motif recovery rates. In other words, compared to both MCL’s clusters and AP’s clusters, the motif recovery rate of CLIMP’s clusters is becoming more highly saturated after the 130th cluster, and the CLIMP’s clusters that contain known motifs rank higher on the sequence of the top 130 clusters than do MCL’s and AP’s. Clearly, the top ranked clusters contain more known motifs than low ranked ones. Note that those clusters that do not contain any known motif might be novel ones. Specially, CLIMP’s clusters essentially achieve the saturated condition in the 200th cluster, which is consistent with the number of transcription-related proteins in the DBD database [49] and two references [50, 51]. Furthermore, Fig 6(B) shows that CLIMP’s clusters contain less cumulative putative motifs than AP’s and MCL’s; therefore, CLIMP can filter out more spurious motifs than the other clustering algorithms.

### Performance on clustering motifs for ChIP datasets

In DePCRM [13], which is a tool for *de novo* prediction of *cis*-regulatory elements (CREs) and modules from ChIP datasets in an eukaryote, 168 ChIP datasets of 56 TFs from *Drosophila melanogaster* were collected from the Berkeley drosophila transcription network project (BDTNP) [52], the modENCODE project [53], and literature. The majority of the binding peaks in these datasets have a length of around 1,000 bp. In the binding peaks of each ChIP dataset, DREME [54] was selected in DePCRM to identify all possible motifs. Finally, a total of 17,890 putative motifs containing 35,359,819 putative CREs were identified in 162 datasets of the 168 ChIP datasets (6 low-quality datasets were removed). Clearly, the vast majority of the putative motifs found in the datasets are spurious predictions. The TOMTOM motif comparison tool (http://mccb.umassmed.edu/meme/cgi-bin/tomtom.cgi) was used to compare putative motifs with the known motifs of *D*. *melanogaster* in the Redfly v3.0 [55], FlyFactorSurvey [56] and FlyReg [57] databases. For each of the 17,890 putative motifs, we say it is likely a true motif if it is highly similar to known motifs in *D*. *melanogaster* at *p*<0.001. After doing the comparisons using TOMTOM, we found that the 17,890 putative motifs cover 144 known true motifs of *D*. *melanogaster* with *p*<0.001.

**Fig 7:**
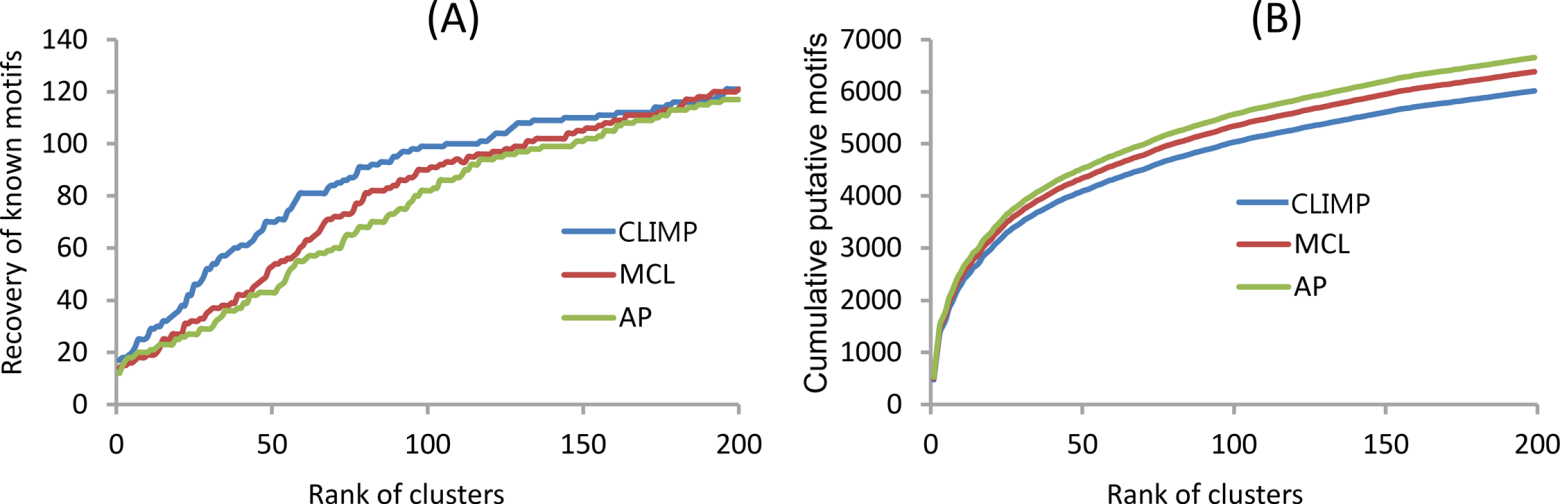
Evaluation of the three clustering algorithms in a ChIP dataset. Cumulative numbers of recovered known motifs (A) and putative motifs (B) of the ChIP datasets in the top-ranked clusters produced by MCL, AP, and CLIMP, respectively.

Similar to the first experiment, MCL, AP, and CLIMP are tested to cluster the 17,890 putative motifs to see whether or not the clusters that hit known true motifs rank high on the cluster list. At first, we construct a motif similarity graph using the putative motifs as nodes and linking any two motifs by an edge if their SPIC metric score is no less than a preset cutoff γ. Based on the analysis in the first experiment, the motif similarity score cutoff γ is set as 0.6, and the three clustering algorithms with the parameters that are the same as in the first experiment (i.e., the Inflation parameter value of MCL is 2.6, the Reference parameter value of AP is 0.55, and (*α*, *β*) = (0.5, 0.5) for CLIMP) are successively run on the resulting motif similarity graph. As shown in Fig 7(A), in most cases (up to the top 160 ranked clusters), CLIMP cumulatively recovers more known motifs than AP and MCL. Furthermore, as shown in Fig 7(B), CLIMP’s ranked clusters contain less cumulative putative motifs than AP’s and MCL’s. Consequently, CLIMP can filter out more spurious motifs than the other two clustering algorithms.

### Computational speeds

Without parallel computing design, MCL is the fastest program among the three evaluated clustering algorithms. For sparse or small graphs, the running times of the three algorithms are acceptable. Since there is not an available parallel version of AP, the computational speeds of CLIMP are compared to MCL on a workstation with Intel Xeon E5 CPUs. When CLIMP and MCL were run on the aforementioned graph with 145,581 nodes and 34,413,340 edges (the similarity score cutoff was set as 0.6) in the section of evaluating the yeast phylogenetic foot-printing dataset, MCL requires three hours wall-clock time with one thread; in contrast, CLIMP requires twelve hours wall-clock time with one thread, and its running time is reduced to about three hours if ten processes are called. Therefore, it is necessary for CLIMP to speed up by parallelizing its program.

**Fig 8:**
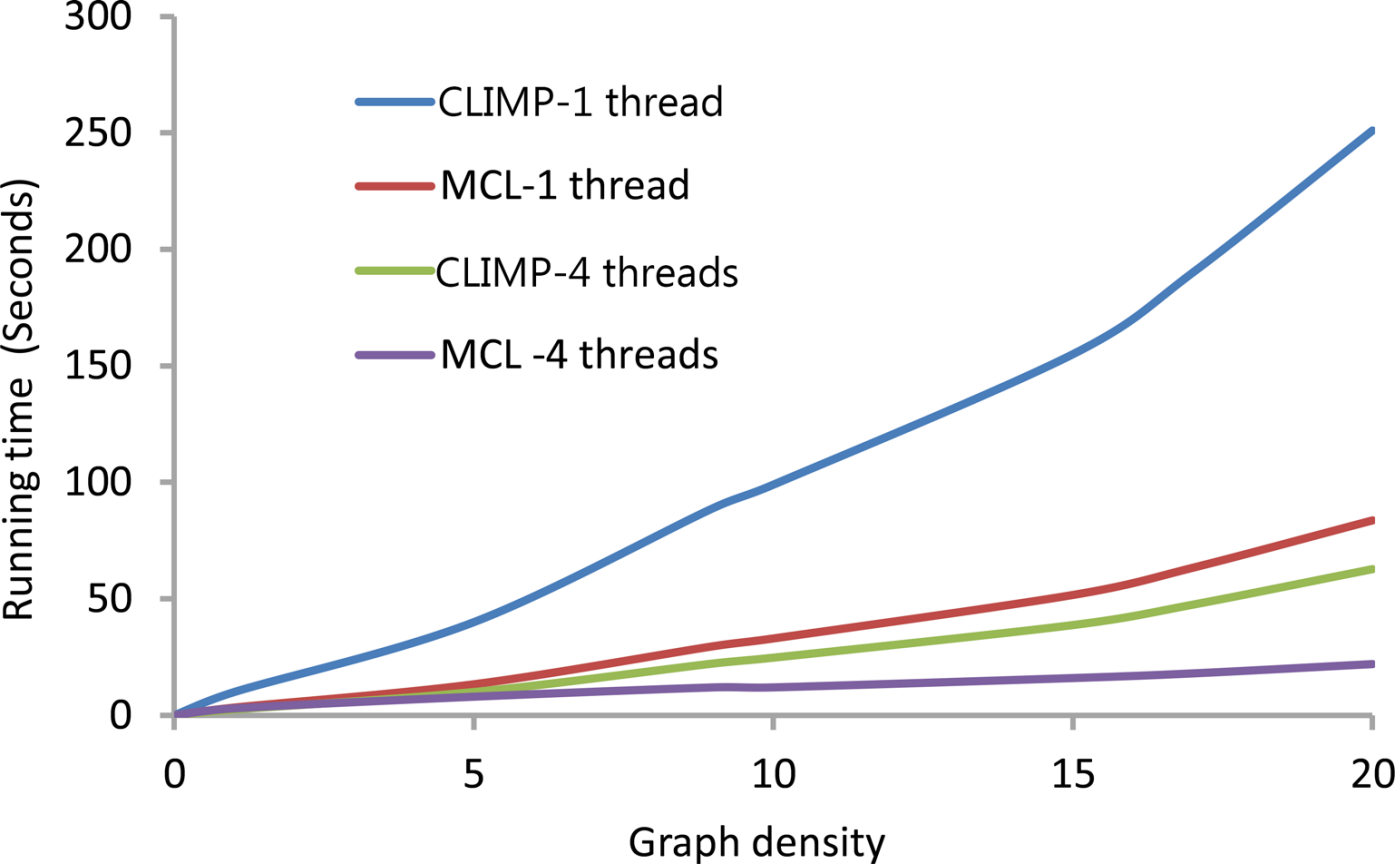
Running time statistics. The running times of CLIMP and MCL on graphs with different densities with either one or four threads.

For further comparison, 2,000 nodes are randomly selected from the 145,581 nodes (motifs) in the section of the yeast dataset when different similarity score cutoffs were selected from 0.10 to 0.95 in steps of 0.05, so a series of motif similarity graphs are constructed with different graph densities (the density of a graph is defined as the number of edges divided by the number of nodes). Single process and four processes are called respectively by CLIMP and MCL on these constructed graphs with different densities. The running times are plotted in Fig 8, which shows that CLIMP’s running time is acceptable if enough processes (threads) are called; however, in most cases, CLIMP is slower than MCL because CLIMP is a heuristic enumeration algorithm with time complexity $O(|V|\cdot \max_{v\in V}\{d_{v}^{2}\})$ while MCL is a stochastic flow simulation algorithm with time complexity *O*(|*V*|^2^). If a graph is very sparse, CLIMP runs faster than MCL. But if the graph is dense, MCL runs faster than CLIMP, but the computational speed of CLIMP can be improved by using more computer nodes.

### Conclusions and Availability

In the paper, a new efficient clustering algorithm is proposed for large-scale motif clustering, which can be a complement of MCL and AP in some genome-wide motif prediction pipelines such as GLECLUBS [28], eGLECLUBS [29], and DePCRM [13]. The C++ source code parallelized with openMP, the three datasets used in this article, and a web server of CLIMP are publicly available at http://sqzhang.cn/climp.html.
